# The Association between the Oral-Gut Axis and the Outcomes of Severe COVID-19 Patients Receiving Extracorporeal Membrane Oxygenation: A Case-Control Study

**DOI:** 10.3390/jcm11051167

**Published:** 2022-02-22

**Authors:** Aya Yoshino, Yoshihiko Nakamura, Shiho Hashiguchi, Shintaro Ishida, Ryosuke Mano, Shinsuke Nakamura, Ryosuke Kita, Mika Seto, Tohru Takata, Hiroyasu Ishikura, Seiji Kondo

**Affiliations:** 1 Department of Oral and Maxillofacial Surgery, Faculty of Medicine, Fukuoka University, 7-45-1 Nanakuma, Jonan-ku, Fukuoka 814-0180, Japan; shpon19901222@gmail.com (S.H.); shintarou19910118@gmail.com (S.I.); md180027@cis.fukuoka-u.ac.jp (R.M.); nakamura0506@fukuoka-u.ac.jp (S.N.); rkita@fukuoka-u.ac.jp (R.K.); miichan@fukuoka-u.ac.jp (M.S.); kondo@fukuoka-u.ac.jp (S.K.); 2 Department of Emergency and Critical Care Medicine, Faculty of Medicine, Fukuoka University, 7-45-1 Nanakuma, Jonan-ku, Fukuoka 814-0180, Japan; pdmxy827@yahoo.co.jp (Y.N.); ishikurah@fukuoka-u.ac.jp (H.I.); 3 Department of Oncology, Hematology, and Infectious Disease, Fukuoka University Hospital, 7-45-1 Nanakuma, Jonan-ku, Fukuoka 814-0180, Japan; takattol@cis.fukuoka-u.ac.jp

**Keywords:** oral–gut axis, COVID-19, mortality

## Abstract

The novel conceptual disease model, the oral–gut axis, which represents the immunomodulatory mutual relationship between oral and gut microbial compartments, has been attracting attention in relation to systemic health issues. We investigated whether this unique crosstalk influences the systemic condition of patients with COVID-19 infections who received extracorporeal membrane oxygenation (ECMO) in the intensive care unit (ICU) during April and December 2020. In this case-control study, patients were divided into two groups according to their survival (total entry size, *n* = 21; survivors, *n =* 13; non-survivors, *n =* 8). Patients were evaluated using the oral assessment guide from Fukuoka University (OAG-F) and the Bristol Stool Form Scale (BSFS) to examine the oral and fecal conditions. A blood-based inflammatory factor, the neutrophil-to-lymphocyte ratio (NLR), was used as an indicator of systemic immunity. The high total OAG-F scores were associated with both elevated BSFS and NLR values, and a mutually positive correlation between BSFS and NLR was observed. This indicated an interplay between oral deterioration, gut dysbiosis, and the impairment of immunity. Furthermore, oral deterioration was more frequently observed in non-survivors on day 14 of ICU admission. In addition, on days 7 and 21 of ICU admission, impaired immunity, reflected by an elevated NLR, was observed in non-survivors. However, the distribution of the gut microbiome—reflected by increased BSFS values—with the time it was examined was not directly observed in non-survivors. Taken together, these findings suggested that oral–gut health may be specifically associated with mortality in COVID-19 patients receiving ECMO in the ICU.

## 1. Introduction

Periodontal diseases are the most prevalent and chronic inflammatory oral diseases; they occur in response to a dysbiosis in the subgingival biofilm [1,2]. *Porphyromonas gingivalis*, a representative of the periodontopathogens, plays a critical role, not only in disease initiation and progression, but also in the onset of various systemic pathologies [3]. On the appearance of inflammation in the subgingival pockets, periodontopathogens can invade the ulcerated sulcular epithelium and can then disseminate into the systemic circulation. In addition, systemic inflammation comes with modest increases in the levels of inflammatory cytokines, such as tumor necrosis factor-α, interleukin-1β, IL-4, IL-6 and IL-10. Therefore, periodontal diseases have been considered to be immune-mediated, and have a mutual association with several chronic inflammatory systemic diseases, including diabetes and cardiovascular diseases, through the facilitation of these inflammatory mediators and/or the hematogenous dissemination of oral bacteria [1,4,5]. Dysbiotic oral microbiota may translocate to the respiratory/gastrointestinal tracts, reducing diversity and causing a shift in the bacterial composition of these distal organs, leading to an impairment of physiological systemic homeostasis, primarily in metabolism and in immunity. This theory has recently received increased attention as a novel conceptual disease model: the oral–gut axis [6,7,8,9]. Indeed, it has already been reported that oral pathobionts, such as the *Klebsiella/Enterobacter* species and *Fusobacterium nucleatum* can ectopically colonize the intestine, leading to the promotion of colitis [10] and the progression of colorectal cancer [11,12], respectively.

COVID-19, which is caused by the novel coronavirus, the severe acute respiratory syndrome coronavirus 2 (SARS-CoV-2), can trigger respiratory failure, followed by multiple organ failure, which is characterized by elevated blood levels of inflammatory cytokines/chemokines: the so-called cytokine storm syndrome. These exacerbating excessive inflammatory reactions are associated with the complications of COVID-19 through the mutual interaction with several of the above-described comorbidities, including diabetes, cardiovascular disease, and periodontal disease [6,13]. In some severe cases, intensive care unit (ICU) admission for oxygen supplementation and mechanical ventilation (other than extracorporeal membrane oxygenation (ECMO), which is reserved as a last resort to restore respiratory function) are required as a direct result of life-saving therapies [14].

With regard to the crosstalk between the gut microbiome and the viral infection, hospitalized COVID-19 patients have significant alterations in their fecal microbiome [15,16], and gut microbiome dysbiosis may predispose patients to the severe consequences of COVID-19 [6,17]. In contrast, since SARS-CoV-2 enters the body via the oral cavity and oropharynx, where epithelial cells that express the angiotensin-converting enzyme-2 (ACE2) and transmembrane protease serine 2 (TMPRSS2) virus receptors are found [18], critical mutual interactions between the resident oral microbiome and the virus exist, which favors the oral dysbiosis and/or the establishment of the viral infection and disease progression [1,4,5]. Recently, we reported that poor oral health was associated with mortality in COVID-19 patients receiving ECMO in the ICU, although the duration of ECMO was not directly related to mortality [19]. However, whether the potential impact of the immunomodulatory functions via the interaction between the oral and gut microbiomes—the oral–gut axis—is associated with the severity of COVID-19 symptoms remains to be elucidated.

In this study, we investigated the relevance of this axis in a case-control study of severe COVID-19 patients receiving ECMO in the ICU.

## 2. Materials and Methods

### 2.1. Study Design

The present study included 21 consecutive inpatients who were diagnosed with COVID-19 in the Fukuoka University Hospital ECMO Center between April and December 2020. The patient group contained two patients who were hospitalized at the Center at the end of 2020, in addition to the patients previously reported [19]. The following data were collected: sex, age, and the length of the stay in the Center. We investigated correlations using data points among the oral assessments, stool assessments, and blood tests, which were performed on the same day.

### 2.2. Oral Assessment

Oral assessments were carried out, as described previously [19]. The oral health status of all eligible patients was evaluated on the first day of admission by nurses, according to the oral assessment guide from Fukuoka University (OAG-F); thereafter, each patient received an oral assessment every day.

### 2.3. Oral Health Care

Oral health care was carried out, according to a previously described protocol [19]. Briefly, all patients received continuous oral care, which consisted of cleaning the oral mucosa and tongue using a sponge brush 3–8 times a day.

### 2.4. Stool Samples

An active surveillance culture was performed using stool samples once a week until the patient left the ECMO Center. The fecal condition was estimated according to the Bristol Stool Form Scale (BSFS) category, which classifies samples into seven types, with the highest scores corresponding to loose, liquid stools, while lower scores correspond to hard stools [20]. An elevated BSFS value suggests an impairment of the gut microbial community [21], thus leading to the development of a number of diseases in the gastrointestinal tract [22].

### 2.5. Index of the General Condition

Since exacerbating excessive inflammatory reactions are a hallmark of severe COVID-19, we considered the use of inflammatory parameters. A blood-based inflammatory parameter, the neutrophil-to-lymphocyte ratio (NLR), was used. This is calculated as the absolute neutrophil count divided by the absolute lymphocyte count [23]. Although patients with SARS-Cov-2 infections sometimes show a significant reduction in the number of T-cells [24], we used this score as an indicator of systemic immunity because this score has been used to predict mortality in HIV-infected patients, who, similarly, show decreased T-cell counts (CD4^+^ counts) [25,26].

### 2.6. Statistical Methods

The correlations between the total OAG-F Score, the BSFS, and the NLR were determined using Spearman’s rank correlation coefficient.

To assess the efficacy of factors while considering the time course, patients were grouped as survivors and non-survivors. The statistical comparisons of the two groups were performed at comparable time points (at admission, day 7, day 14, and day 21), to take the duration of the ICU stay into account. Their results were then compared using Fisher’s exact test, the Mann–Whitney U-test, and Wilcoxon’s signed-rank test. *p*-values of less than 0.05 were considered to demonstrate a significant difference.

Data were analyzed using JMP version 14.0 (JMP Institute, Tokyo, Japan).

### 2.7. Ethical Considerations

The study was approved by the Clinical Research and Ethics Centre of Fukuoka University (No. U21-03-006). This research was conducted under the same approval as the approval described in our previous report [19].

## 3. Results

### 3.1. Characterics of COVID-19 Patients Who Received ECMO

Of the 21 patients enrolled in the study, a total of 229 data points were investigated to analyze any correlations, which included oral assessments, stool assessments, and blood tests, which were performed on the same day.

Table 1 shows the baseline characteristics of the study subjects (80.1% male; median age, 62 years; interquartile range (IQR), 54–69 years). Thirteen of the patients survived, and eight died. The age and the length of stay in the center did not differ to a statistically significant extent (*p* > 0.05).

### 3.2. Correlations among the Total OAG-F, NLR, and BSFS Scores

**Figure 1:**
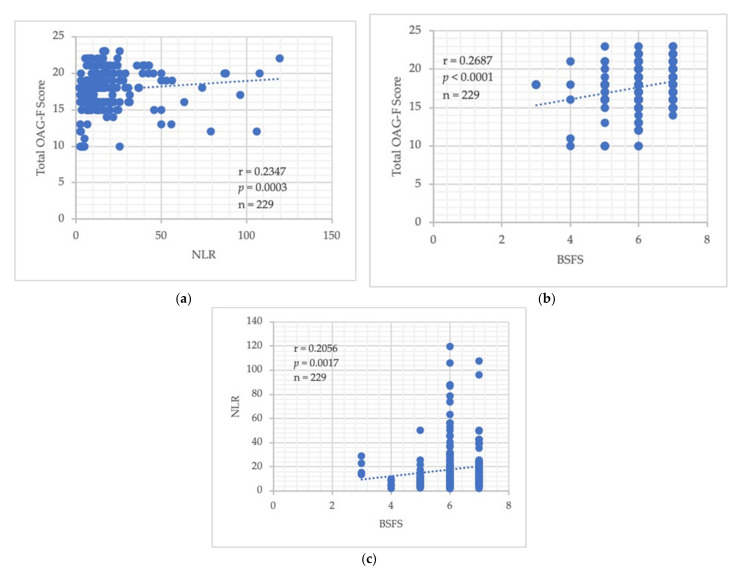
Pearson’s correlation coefficients (r) between (**a**) the total OAG-F score and NLR; (**b**) the total OAG-F score and BSFS; (**c**) the NLR and BSFS scores. The blue round circles indicate the individual data points, while the blue dotted lines represent the approximate line.

Of the 229 data points for the 21 patients, high OAG-F scores were slightly associated with elevated NLR and BSFS scores (Figure 1a,b). Furthermore, elevated NLR and BSFS values also showed weak, but valid, correlations with each other (Figure 1c).

### 3.3. The Time Course of the Changes of the Scores of Survivors and Non-Survivors

**Figure 2:**
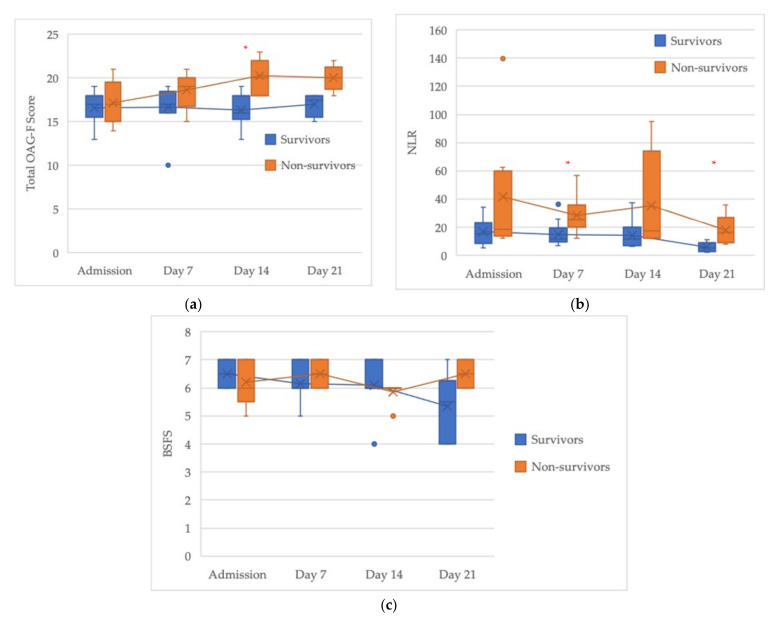
The time course of changes in the scores. (**a**) the total OAG-F score. (**b**) the NLR. (**c**) the BSFS score. The boxplots show the median (x mark), IQR (boxes), and total range (whiskers). The blue and orange circles indicate outliers. * Asterisks indicate a statistically significant difference (*p* < 0.05).

We compared the survivors and non-survivors at equal time-points during their ICU stay. Figure 2 shows the change in the treatment time course in the total OAG-F, NLR, and BSFS scores. On day 14, the total OAG-F score of the survivors was significantly higher than that of non-survivors (*p* < 0.05) (Figure 2a). On days 7 and 21, the NLRs of non-survivors were significantly increased in comparison to survivors (*p* < 0.05) (Figure 2b). In contrast, the BFSF values of survivors and non-survivors did not differ to a statistically significant extent at any point in the time course (Figure 2c).

## 4. Discussion

The connected anatomy beginning at the oral cavity and ending at the gut composes the gastrointestinal tract as the digestive system, and distinctive microbiota are harbored along this tract. When oral microbiome dysbiosis occurs, the oral bacteria potentially translocate into the gut following the disruption of the resident microbiome in the intestine, leading to the promotion of immune dysregulation via the modulation of several innate and adaptive immune regulatory pathways [7]. Indeed, oral bacteria migrate into the lower gastrointestinal tract in healthy people. Of note, increased oral-to-gut microbial transmission is observed in several diseases, including rheumatoid arthritis [27] and colorectal cancer [12], in comparison to healthy individuals, and the increased presence of specific oral bacteria in the intestine has been reported to contribute to the progression of these diseases [28]. This clinical concept of the oral–gut axis has attracted great interest as a novel disease model. The previous findings have been bacteriologically and genetically analyzed, and the studies have a high level of evidence. However, conversely, the association of this concept with the impact of a COVID-19 infection, especially in ECMO patients, has remained largely unexplored.

In this study, high OAG-F scores, which imply oral deterioration, were associated with both elevated NLRs and BSFS scores (Figure 1a,b), which reflect impaired immunity and the impaired distribution of the gut microbiome, respectively. Synergistically, there was a positive correlation between the NLR and BSFS values (Figure 1c), suggesting an intimate link between oral and gut dysbiosis; that is, a poor oral–gut axis was present, accompanied by immune perturbations. Furthermore, oral deterioration was more frequently observed in non-survivors on day 14 of admission to the ICU (Figure 2a). Simultaneously, the immune dysregulation reflected by the elevated NLR was also observed in non-survivors on days 7 and 14 (Figure 2b), resulting in a vicious circle with the progression of time. A difference in the extent of gut dysbiosis was not observed between the two groups during the time period that was examined (Figure 2c). Possible explanations for this include malnutrition, an increased susceptibility to treatment-related toxicity due to the rapid turnover cycle of the intestinal mucosa in both survivors and non-survivors, and the fact that the overall gut microbiome was unstable in a subset of patients with COVID-19 during the period of hospitalization [15]. Furthermore, enteral nutrition might be poorly tolerated during the duration of ECMO treatment in the ICU.

The present study was associated with some limitations. Most importantly, the relatively small number of patients did not allow us to perform comprehensive multivariable analyses. In addition, there was some variability in the treatment regimens of the two groups, including pharmacotherapy (antibiotics, steroids, etc.), nutrition therapy, and the duration of orotracheal intubation and ECMO. Further prospective studies should be undertaken to analyze severe cases without ECMO as a control group. Furthermore, we did not provide direct evidence demonstrating that the oral cavity-derived pathobionts, showing immune-stimulatory activity, exist in the feces of patients with COVID-19 by means of metagenomics sequencing. There is no evidence that greater amounts of oral-to-gut microbial transmission was observed in COVID-19 patients who received ECMO. Nevertheless, there is, at least in part, a correlation of coexisting oral dysbiosis and gut abnormalities with the disease course and prognosis of COVID-19 patients receiving ECMO. The initial identification of COVID patients, with the coexistence of oral dysbiosis and gut abnormalities, could help to predict the outcomes of severe COVID patients and could lead to the development of more effective treatments that combine routine oral hygiene intervention and symbiotic therapy [29,30]. Therefore, a multidisciplinary approach should be strongly promoted by emergency physicians, as well as gastroenterologists, oral healthcare specialists, and nurses integrated into ICU teams.

## 5. Conclusions

The present findings suggest that it is plausible that the oral–gut axis, across the two major organs, affects the immunocompromised host and mortality rates in severe COVID-19 patients receiving ECMO, thus supporting the possibility that improving oral–gut health may reduce the risk of complications from COVID-19. Further investigations should be performed to investigate the association of oral–gut health with the severity of COVID-19 infections in the ICU.

## Figures and Tables

**Table 1:** Patient characteristics.

		All Cases N = 21	Survivors N = 13	Non-Survivors N = 8
Sex ^1^	Male	17 (81.0%)	11 (84.6%)	6 (75.0%)
	Female	4 (19.0%)	2 (15.4%)	2 (25.0%)
Age ^2^		62 (54–69)	58 (43–64)	67 (60–71)
Length of stay in the center (days) ^2^		23 (12–46)	17 (11–31)	34 (17–74)

^1^ Value represents the number (%); ^2^ Value represents the median (IQR).

## Data Availability

Not applicable.
